# Efficacy and safety of Jinhuang ointment in treating acute gouty arthritis: a retrospective cohort study

**DOI:** 10.3389/fphar.2025.1728414

**Published:** 2025-12-11

**Authors:** Honglin Wang, Huiling Mei, Yuyu Duan, Wei Hua, Aoshuang Xu, Lin Lu

**Affiliations:** 1 Department of Orthopedics, Renmin Hospital of Wuhan University, Wuhan, China; 2 Department of Rheumatology and Immunology, Union Hospital, Tongji Medical College, Huazhong University of Science and Technology, Wuhan, China; 3 College of Chinese Medicine, Hubei University of Chinese Medicine, Wuhan, China; 4 Institute of Hematology, Union Hospital, Tongji Medical College, Huazhong University of Science and Technology, Wuhan, China

**Keywords:** gouty arthritis, Jinhuang ointment, traditional Chinese medicine (TCM), gout, retrospective study

## Abstract

**Objective:**

Non-steroidal anti-inflammatory drugs (NSAIDs) for acute gout often carry risks of gastrointestinal adverse effects and hepatic, renal, and cardiovascular toxicity. To explore safer treatment options and reduce the use of these drugs and associated risks, this study aimed to evaluate the clinical efficacy and safety of external application of Jinhuang Ointment in the treatment of acute gouty arthritis, providing evidence for optimizing clinical management strategies.

**Methods:**

A total of 104 patients with acute gouty arthritis (July 2023–June 2024) were retrospectively assigned to three groups: the control group (n = 35) received conventional Western therapy (celecoxib, low-purine diet, >2000 mL daily water intake, and sodium bicarbonate); T1 group (n = 35) received conventional therapy plus topical diclofenac diethylamine emulsion; T2 group (n = 34) received conventional therapy plus Jinhuang Ointment. All patients completed 7-day treatment. Baseline data including demographic characteristics, gout history, serum uric acid levels, onset time, and target joint distribution were collected. Primary outcome indicators, including pain improvement, joint tenderness, swelling, and joint mobility in the target joint. Secondary outcomes covered changes in serum uric acid levels and inflammatory markers (CRP, ESR). Adverse events occurring during the treatment period were also documented.

**Results:**

Baseline characteristics were comparable across groups. After treatment, all groups showed significant improvements in VAS scores, joint swelling, tenderness, and mobility (P < 0.05). The T2 group exhibited a significantly shorter time to pain improvement compared to both the T1 and control groups (P < 0.05). Both T1 and T2 groups showed better outcomes in joint tenderness, swelling, and joint mobility than the control group (P < 0.05), though only T2 demonstrated significant superiority in swelling reduction (P < 0.05). Laboratory results indicated that CRP and ESR decreased more markedly after treatment in T1 and T2 than in controls (P < 0.05), with no intergroup difference in uric acid reduction. Adverse event rates were similar among groups, with no significant differences observed.

**Conclusion:**

Jinhuang Ointment combined with conventional therapy significantly improves joint pain and swelling, accelerates symptom relief, and exhibits particular efficacy in swelling reduction, without increasing safety risks. Its multi-component, multi-target mechanism provides a potential explanation for the clinical efficacy observed, thereby supporting the need for higher-quality studies to further verify its application value.

## Introduction

Gout is a clinical syndrome caused by genetic or acquired factors that disrupt purine metabolism (Dehlin et al., 2020; Singh and Gaffo, 2020). This metabolic disturbance leads to the overproduction and/or impaired excretion of uric acid, ultimately resulting in the deposition of monosodium urate crystals in joints or other tissues (Im et al., 2025). In recent years, alongside economic development and lifestyle changes, the global prevalence and incidence of gout have continued to rise, with a notable trend toward earlier onset (Kuo et al., 2015). Acute gouty arthritis (AGA) is the most common initial manifestation and fundamental type of gout (Ragab et al., 2017). Its pathological basis involves intense inflammatory reactions triggered by the deposition of monosodium urate crystals, clinically characterized by joint redness, swelling, heat, pain, and functional impairment, all of which significantly impact patients' quality of life (Dalbeth et al., 2021; VanItallie, 2010).

Current conventional treatments during the acute phase focus primarily on rapid anti-inflammatory and analgesic effects. First-line medications commonly include nonsteroidal anti-inflammatory drugs (NSAIDs), colchicine, and glucocorticoids (Zhang, 2023). Although these agents can relatively quickly control symptoms, they are frequently associated with adverse effects such as gastrointestinal irritation, hepatorenal impairment, increased risk of cardiovascular events, and hormone-related metabolic disturbances (McKenzie et al., 2021; Vargas-Santos et al., 2019; Choi et al., 2018). Moreover, symptom rebound frequently occurs after discontinuation, limiting their clinical utility (Bardin and Richette, 2017). Additionally, the frequent presence of comorbidities in gout patients further complicates medication management and increases therapeutic risks (Ragab et al., 2017; Choi et al., 2022). Therefore, there is a pressing clinical need to explore complementary and alternative therapies that can effectively alleviate acute symptoms while reducing drug-related side effects.

In Traditional Chinese Medicine (TCM), gout is classified under categories such as “Bi Zheng” (impediment syndrome) and “Li Jie Feng” (joint-running wind) (Chi et al., 2020). The Yuan Dynasty physician Zhu Danxi first established the disease name “Tong Feng” and accumulated extensive formulary experience (Gao and Zhao, 2018), with remedies such as Simiao Pills and Shangzhongxia Tongfeng Formula demonstrating significant effects in improving inflammatory symptoms and reducing inflammatory markers (Pu et al., 2021). External treatment with Chinese herbal medicine, as an important component of traditional medicine, is widely used in clinical practice due to its simple application, direct topical action, and avoidance of first-pass hepatic metabolism (Huang et al., 2019). Modern research has confirmed that active components in topical herbal preparations can be transdermally absorbed through the skin into the lymphatic and circulatory systems, thereby exerting pharmacological effects (Hwang and Song, 2021). The TCM theory of “transdermal absorption” further elucidates how medicinal substances, through meridian conduction, can reach internal organs and connect with interstitial spaces, thereby improving local microcirculation, promoting inflammation resolution, and providing a theoretical basis for the external use of Chinese medicine in treating AGA (Tian, 2019).

Currently, external treatment with Traditional Chinese Medicine has demonstrated promising application prospects in the management of AGA. Several classic topical preparations, such as Qingpeng Ointment (Shang et al., 2024), Qingluo San (Shu et al., 2022), and Qingbi Granules (Ren et al., 2020), are widely used in clinical practice and have demonstrated satisfactory efficacy in gout patients with damp-heat obstruction syndrome. Growing evidence not only confirms their clinical value but also begins to increasingly clarifies their therapeutic profiles through more rigorous study designs. For instance, a double-blind, placebo-controlled trial demonstrated that Qingpeng Ointment was superior to placebo in relieving joint pain and swelling, improving joint function, with a favorable safety profile (Shang et al., 2024). Ren et al. (2020) found that the external application of Qingbi Granules combined with conventional Western medicine more effectively improved joint pain and swelling, shortened the duration of NSAID use, and reduced levels of C-reactive protein (CRP) and serum uric acid. Shu et al. (2022) reported that the external use of Qingluo San combined with diclofenac sodium dual-release enteric-coated capsules provided faster pain relief and demonstrated advantages in resolving joint swelling and improving functional mobility. However, while these studies confirm the overall value of TCM external treatments, Jinhuang Ointment—another classic formula known for its heat-clearing, detoxifying, anti-inflammatory, and analgesic properties (Ye et al., 2022)—has not yet been adequately evaluated within a similarly rigorous research framework. Specifically, for a classic compound formula such as Jinhuang Ointment, which consists of multiple herbs carefully combined according to the 'sovereign, minister, assistant, and courier' (Jun-Chen-Zuo-Shi) principle, existing studies often remains at the stage of preliminary clinical validation (Ye et al., 2022). There is a lack of rigorous retrospective or prospective cohort studies systematically evaluating its comprehensive efficacy (e.g., symptom scores, TCM syndrome efficacy) and safety (e.g., dermal adverse reactions, hepatorenal function) in real-world clinical settings. In particular, the synergistic effects and potential risks of its combination with conventional Western medicine have not been adequately defined.

Therefore, a systematic evaluation of Jinhuang Ointment—an empirical formula used clinically for nearly five decades—is warranted. The formula comprises Rhei Radix et Rhizoma (Dahuang), Phellodendri Chinensis Cortex (Huangbai), Arisaema Cum Bile (Nanxing), Curcumae Longae Rhizoma (Jianghuang), Angelicae Dahuricae Radix (Baizhi), Citri Reticulatae Pericarpium (Chenpi), Atractylodis Rhizoma (Cangzhu), Magnoliae Officinalis Cortex (Houpo), Trichosanthis Radix (Tianhuafen), and Glycyrrhizae Radix et Rhizoma (Gancao). In this formula, Rhei Radix et Rhizoma and Phellodendri Chinensis Cortex serve as sovereign herbs to clear heat and detoxify; Angelicae Dahuricae Radix, Curcumae Longae Rhizoma, and Trichosanthis Radix act as minister herbs to reduce swelling, relieve pain, astringe sores, and disperse nodules; Atractylodis Rhizoma, Magnoliae Officinalis Cortex, Citri Reticulatae Pericarpium, and Arisaema Cum Bile serve as assistant herbs to dry dampness, regulate qi, and reduce swelling. Glycyrrhizae Radix et Rhizoma harmonizes the formula and also clears heat. Together, these components achieve the effects of clearing heat, cooling the blood, resolving stasis, and relieving pain, making the formula particularly suitable for acute gouty arthritis of the damp-heat accumulation type. While modern pharmacological studies have begun to reveal its mechanisms of action (Li et al., 2023), higher-level clinical trials are essential to establish its true therapeutic value, especially in combination with Western medicine.

Therefore, this study aims to systematically evaluate the clinical efficacy and safety of Jinhuang Ointment combined with conventional Western medicine in the treatment of acute gouty arthritis through a retrospective cohort study, with the goal of providing more reliable evidence for integrative TCM and Western medicine practice.

## Materials and methods

Using the hospital HIS system, we retrieved medical records of patients diagnosed with acute gouty arthritis who received treatment between July 2023 and June 2024 from the Department of Orthopedics at Renmin Hospital of Wuhan University and the Department of Rheumatology at Wuhan Union Hospital. Search keywords included “gout,” “hyperuricemia,” and “gouty arthritis.”

### Data anonymization

To protect patient confidentiality, all extracted data were anonymized immediately. This involved the permanent deletion of all direct identifiers, including patient names, hospital admission numbers, and national ID numbers. Each patient was subsequently assigned a unique, non-traceable study ID to ensure complete de-identification in accordance with ethical standards.

### Data extraction and quality control

A rigorous process was implemented for data extraction and verification. Two researchers independently extracted data from the electronic medical records, followed by a cross-verification procedure. Any discrepancies identified were resolved through consultation with a third senior researcher or by re-examining the original medical records. Furthermore, logical consistency checks were performed on key variables. For instance, we verified that the recorded “time to pain improvement” did not exceed the “total length of hospital stay.”

Moreover, a predefined protocol was established for managing missing data. Cases with missing critical data, such as primary outcomes (e.g., VAS scores or serum uric acid levels), were excluded from the analysis based on predefined exclusion criteria. For non-critical variables with sporadic missing entries, the occurrence was documented, and the missing status was appropriately accounted for during the statistical analysis.

### Study group assignment

Retrospective grouping was based on the actual treatment selection at the time of the patient’s clinic visit. Specifically, the assigned treatment group was determined by the physician after comprehensive consideration of factors such as the severity of the patient’s condition, their response to prior treatments, and their acceptance of different types of topical medications (e.g., whether they had any aversion to traditional Chinese herbal ointments). Based on the treatment method,the patients were categorized into a control group and two distinct treatment groups.

### Diagnostic criteria

#### Diagnostic criteria of AGA

The diagnosis was established with reference to the “2015 American College of Rheumatology/European League Against Rheumatism (ACR/EULAR) Gout Classification Criteria” (Zhang et al., 2017).

#### Differentiated criteria of the dampness-heat syndrome

The diagnosis was established according to the criteria for gout of the dampness-heat accumulation pattern outlined in the Criteria for Diagnosis and Therapeutic Effects of Diseases and Syndromes in Traditional Chinese Medicine (Medicine SAoTC, 1994), manifesting as joint redness, swelling, warmth, and pain with sudden severe onset and limited mobility, systemic symptoms including fever, irritability and thirst, a red tongue with yellow-greasy or yellow-thick coating, and a wiry-slippery or slippery-rapid pulse.

### Inclusion criteria

1. Meeting the aforementioned diagnostic criteria of both traditional Chinese and Western medicine with a pain score ≥3 points
2. No prior use of other relevant medications or alternative treatments after the onset of gout
3. Age between 18 and 65 years, regardless of gender
4. Interval between the last treatment and the current episode <72 h
5. Primary observation sites including the first metatarsophalangeal joint, dorsum of the foot, ankle joint, and knee joint, with only the most severely affected joint (target joint) recorded for each subject and remaining consistent throughout the observation period
6. Patients whose systemic treatment regimen during natural clinical management included one of the following celecoxib alone, celecoxib combined with topical diclofenac diethylamine emulsion application, or celecoxib combined with topical Jinhuang Ointment application
7. Complete clinical data, including records of symptoms, signs, and laboratory examinations before and after treatment

### Exclusion criteria

1. Development of concurrent conditions interfering with normal use of the study medication, or requirement of additional medications affecting treatment efficacy during the research period
2. Presence of severe systemic diseases or serious comorbidities affecting normal treatment implementation
3. Changes in treatment regimen, self-discontinuation of medication, or irregular drug administration resulting in poor compliance
4. Diagnosis with other forms of arthritis (such as rheumatoid arthritis or septic arthritis)
5. Loss to follow-up due to various reasons leading to significant data gaps
6. Initial misdiagnosis or failure to meet all inclusion criteria
7. Incomplete medical records due to any circumstances

### Treatment methods

Patients were retrospectively assigned to one of three groups based on the treatment regimen they received: the control group, the T1 group, and the T2 group. The baseline characteristics of all patients are presented in Table 1. Patients in the control group received only conventional Western-based treatment, which included a low-purine diet, abstinence from alcohol, daily water intake of more than 2000 mL, along with oral celecoxib (60 mg each time, twice daily) and sodium bicarbonate (1 g each time, three times daily). The T1 group received the conventional treatment plus topical application of diclofenac diethylamine emulsion (manufactured by Beijing Novartis Pharma Ltd.). The T2 group received the conventional treatment plus topical application of Jinhuang Ointment.

**TABLE 1:** Comparison of the baseline characteristics among the three groups.

Variable	Control group n = 35	Treatment group1 n = 35	Treatment group 2 n = 34
Age (years, mean ± SD)	46.00 ± 12.00	47.00 ± 10.20	45.00 ± 12.60
Male, n (%)	33 (94.3%)	32 (91.4%)	32 (94.1%)
Patients with previous gout history, n (%)	28 (80.0%)	29 (82.9%)	28 (82.3%)
Uric acid (pre-treatment) (μmol/L, mean ± SD)	474.57 ± 75.993	470.86 ± 68.337	478.59 ± 70.939
Onset time, n (%)			
≤24 h	4 (11.4%)	5 (14.3%)	6 (17.6%)
24–48 h	10 (28.6%)	9 (25.7%)	9 (26.5%)
48–72 h	16 (45.7%)	17 (48.6%)	16 (47.1%)
Drink, n (%)	12 (34.3%)	13 (37.1%)	12 (35.3%)
Index joint, n (%)			
Metatasophalangeal joint 1	5 (14.3%)	4 (11.4%)	5 (14.7%)
Other joints	6 (17.1%)	6 (17.1%)	8 (23.5%)
Ankle	6 (17.1%)	7 (20.0%)	6 (17.6%)
Knee	5 (14.3%)	8 (22.9%)	6 (17.6%)
Wrist	2 (5.7%)	2 (5.7%)	1 (2.9%)
Hand	3 (8.6%)	3 (8.6%)	3 (8.8%)
Elbow	2 (5.7%)	1 (2.9%)	1 (2.9%)
Multiple joints	4 (11.4%)	2 (5.7%)	2 (5.9%)
Joint swelling, n (%)			
No swelling	0 (0%)	0 (0%)	0 (0%)
Palpable	7 (20.0%)	7 (20.0%)	6 (17.6%)
Visible	22 (61.9%)	21 (60.0%)	21 (61.8%)
Bulging beyond joint margins	6 (17.1%)	7 (20.0%)	7 (20.6%)
Activity, n (%)			
No restricted	1 (2.9%)	1 (2.9%)	1 (2.9%)
Moderate restricted	6 (17.1%)	8 (22.9%)	8 (23.5%)
Significantly restricted	10 (28.6%)	12 (34.3%)	11 (32.4%)
Unbearable, cannot take care of themselves	14 (40.0%)	11 (31.4%)	11 (32.4%)

No significant differences were found in the demographic data of all groups (P all >0.05). Continuous data are presented as mean ± standard deviation (mean ± SD); Categorical data are presented as number (percentage) (n (%)).

Jinhuang Ointment is an internal preparation of our hospital. Its formulation comprises Rhei Radix et Rhizoma (Dahuang), Phellodendri Chinensis Cortex (Yan Huangbai), Magnoliae Officinalis Cortex (Houpo), Trichosanthis Radix (Tianhuafen), Curcumae Longae Rhizoma (Jianghuang), Angelicae Dahuricae Radix (Baizhi), stir-fried Atractylodis Rhizoma (Cangzhu), Arisaema Cum Bile (Yu Nanxing), Glycyrrhizae Radix et Rhizoma (Gancao), and Citri Reticulatae Pericarpium (Chenpi). During preparation, each medicinal material was dried, individually ground, and sieved. They were then stir-fried in a wok until the texture became dry and the color turned yellowish. The processed herbal powder, termed Jinhuang Powder, was mixed with beeswax at a ratio of 1:4 (powder to beeswax) and blended uniformly to form the final ointment. All preparations were produced by the hospital’s pharmacy department in accordance with relevant national standards.

#### Quality control of Jinhuang Powder

The Jinhuang Powder (the powdered form of Jinhuang Ointment used in this study) was prepared and subjected to strict quality control according to our internal“Jinhuang Powder Quality Standard”. This standard was established based on the specifications for powders outlined in the Chinese Pharmacopoeia (2022 Edition). The specific quality control measures included:

1. Preparation Process: All herbal components were pulverized into fine powder, passed through No. 6 sieve (125 μm) to ensure uniform particle size, and thoroughly mixed.
2. Critical Quality Attributes: Each batch of the final product was tested for the following:

Particle Size: 100% of the powder must pass through a No. 6 sieve, and no less than 95% must pass through a No. 7 sieve (95 μm).

Moisture Content: Determined by the drying method, must not exceed 9.0%.

Microbial Limits: Tests for total aerobic microbial count, total combined yeasts and molds count, and the absence of specified pathogens (e.g., *Escherichia coli*) were conducted.

3. Stability data: An 18-month, long-term stability study under ambient conditions confirmed that all tested parameters consistently complied with the quality standard throughout the study period, demonstrating the robustness and reliability of the formulation.

#### Application method for topical agents

The Jinhuang Ointment or diclofenac diethylamine emulsion was applied evenly to the affected area each time, extending 3 cm beyond the edge of the redness, swelling, and pain region. The application thickness was maintained at 4–6 mm, administered twice daily for 4–6 h per application. A 7-day continuous treatment constituted one course. During application, the ointment was covered with non-woven gauze and secured with medical tape to ensure appropriate tightness and firm fixation.

Patients in the control group received only the conventional Western medicine-based treatment. All patients were treated for a total of 7 days.

### Observation indicators

#### Primary clinical outcome indicators

1. Change in VAS score for target joint pain The Visual Analog Scale (VAS) was used to record pain intensity at days 0, 1, 3, and 7 of treatment, with scores ranging from 0 to 10 (0 indicating no pain and 10 indicating unbearable severe pain). Assessments were performed three times daily to ensure consistent evaluation criteria across all groups, and the average value was taken as the daily VAS score. The target joint was defined as the most painful joint reported by the patient at enrollment.
2. Time to onset of pain improvement in the target joint. Pain improvement was defined as achieving a VAS score <3.
3. Comparison of joint tenderness, swelling, and mobility scores before and after treatment among the three groups. Joint tenderness, swelling, and mobility were assessed on day 0 and day 7 of treatment using standardized 3-point scales. Joint swelling was scored as follows: 0 indicated no swelling, 1 indicated palpable swelling, 2 indicated visible swelling, and 3 indicated swelling extending beyond the joint margin. Joint tenderness was graded as: 0 for no tenderness, 1 for mild tenderness, 2 for moderate tenderness accompanied by pain-induced withdrawal upon movement, and 3 for severe tenderness with intolerance to touch. Joint mobility was evaluated using a functional scale where 0 represented normal movement, 1 represented limited mobility without impairment of daily activities, 2 represented significantly restricted mobility preventing general activities but allowing self-care, and 3 represented severe impairment with pain during movement, confinement to bed, and inability to perform self-care.

### Secondary outcome indicators

Laboratory tests including serum uric acid (UA), C-reactive protein (CRP), and erythrocyte sedimentation rate (ESR) were measured before and after treatment.

### Safety evaluation

Vital signs and general physical examinations were assessed and recorded during baseline visits and treatment period visits. Any adverse reactions during treatment in both groups, such as stomach pain/abdominal pain, edema, and skin itching, were documented.

### Post-hoc power analysis

Given the retrospective nature of this study, pre-study sample size calculation was not mandatory as the sample size was passively determined by the availability of eligible medical records in the hospital HIS system. To verify the reliability of the study conclusions and ensure sufficient statistical power, *post hoc* power analysis was performed using G*Power 3.1.9.7 software after data collection and primary outcome analysis.

### Statistical methods

Statistical analysis was performed using SPSS 27.0 software. All continuous variables were tested for normality using the Shapiro-Wilk test. Normally distributed continuous variables were expressed as mean ± standard deviation, with intergroup comparisons conducted using the t-test. For repeated measurement data (e.g., VAS scores at different time points), repeated-measures ANOVA was used for intra-group and inter-group comparisons. If the sphericity test was not satisfied, Greenhouse-Geisser correction was adopted; pairwise comparisons at different time points within the group were performed using Bonferroni correction. For the comparison of non-normally distributed continuous variables among three or more groups, the Kruskal–Wallis H test was first used. If the test results showed statistical significance (P < 0.05), the Mann-Whitney U test was further used for *post hoc* pairwise comparisons, and the Bonferroni method was used for P-value correction. Categorical variables were described as number (percentage), and Fisher’s exact test or the chi-square test was used for intergroup comparisons. p < 0.05 was considered statistically significant.

## Results

### Baseline data

Based on the inclusion and exclusion criteria, a total of 104 patients with acute gouty arthritis were enrolled in this study. They were divided into three groups according to the treatment regimen the control group (35 cases, treated with oral celecoxib), the T1 group (35 cases, treated with oral celecoxib plus topical diclofenac diethylamine emulsion), and the T2 group (34 cases, treated with oral celecoxib plus external application of Jinhuang Ointment). Comparison of baseline characteristics among the three groups, including age, gender, history of gout, pre-treatment serum uric acid levels, time since gout onset, target joint distribution, joint swelling severity, joint mobility limitation, and proportion of alcohol consumption, showed no statistically significant differences (P > 0.05), indicating comparability (Table 1).

In terms of target joint distribution, the affected joints in all three groups were primarily concentrated in the first metatarsophalangeal joint, dorsum of the foot, ankle joint, and knee joint. Involvement of other joints (such as the wrist, hand joints, and elbow) was relatively infrequent, consistent with the clinical characteristic of acute gouty arthritis predominantly affecting lower limb joints. Specifically, the proportion of first metatarsophalangeal joint involvement was 14.3% in the control group, 11.4% in the T1 group, and 14.7% in the T2 group, while knee joint involvement accounted for 14.3%, 22.9%, and 17.6%, respectively. These findings further demonstrate the representative disease profile of the enrolled patients.

### Primary clinical outcome indicators

#### Change in VAS score for target joint pain

Before treatment, no significant differences in VAS scores were observed among the three groups (P > 0.05). Following treatment, all groups exhibited a decreasing trend in scores, with statistically significant differences emerging between groups. A repeated measures ANOVA revealed a significant within-group effect, indicating that VAS scores changed significantly over time across all treatment time points (before treatment, day 1, day 3, and day 7) (F = 2191.350, df_1_ = 3, df_2_ = 303, P < 0.001), consistent with substantial pain relief in all groups over the course of treatment. A significant between-group effect was also observed (F = 6.694, df_1_ = 2, df_2_ = 101, P = 0.002), suggesting that the overall efficacy of the three treatment regimens in relieving pain differed. Further, *post hoc* pairwise comparisons showed no statistically significant differences among the T2 (6.83 ± 1.24), T1 (6.87 ± 1.54), and control (7.03 ± 1.58) groups on day 1 of treatment (P = 0.791). By day 3, the VAS score of the T2 group (3.34 ± 1.20) was significantly lower than that of the control group (4.85 ± 1.55, P < 0.001) and the T1 group (4.00 ± 1.35, P < 0.05). At day 7, the VAS score of the T2 group (1.11 ± 0.60) remained significantly lower than that of the control group (2.27 ± 0.79, P < 0.001). Although the total VAS score reduction over the 7-day period followed a numerical hierarchy (T2, 7.19; T1, 7.18; control, 6.04), the difference between the T1 and T2 groups was negligible, indicating a comparable overall analgesic effect (Table 2).

**TABLE 2:** Comparison of VAS scores between the three groups before and after treatment.

Group	Case	Before treatment	95%CI	After treatment 1 day	95%CI	After treatment 3 days	95%CI	After treatment 7 days	95%CI	Decrease range 7 days after treatment
Control group	35	8.31 ± 1.43	7.815–8.797	7.03 ± 1.58*	6.484–7.567	4.85 ± 1.55*	4.317–5.380	2.27 ± 0.79*	1.996–2.536	6.04
Treatment group 1	35	8.34 ± 1.36	7.874–8.806	6.87 ± 1.54*	6.336–7.359	4.00 ± 1.35*	3.536–4.464	1.16 ± 0.64*	0.941–1.379	7.18
Treatment group 2	34	8.30 ± 1.35	7.825–8.769	6.83 ± 1.24*	6.404–7.267	3.34 ± 1.20*#^	2.918–3.753	1.11 ± 0.60*#	0.902–1.322	7.19
Intra-group	F = 2191.350, df_1_ = 3, df_2_ = 303, P < 0.001									
Inter-group	F = 6.694, df_1_ = 2, df_2_ = 101, P = 0.002									

Continuous data are presented as mean ± standard deviation (mean ± SD); Statistical significant difference between before and after treatment in each group *P < 0.05; Statistical significant difference between treatment group 2 and control group #P < 0.05; Statistical significant difference between treatment group 2 and treatment group 1 ^P < 0.05; df_1_ represents the degree of freedom for the source of variation, and df_2_ represents the error degree of freedom.

**FIGURE 1:**
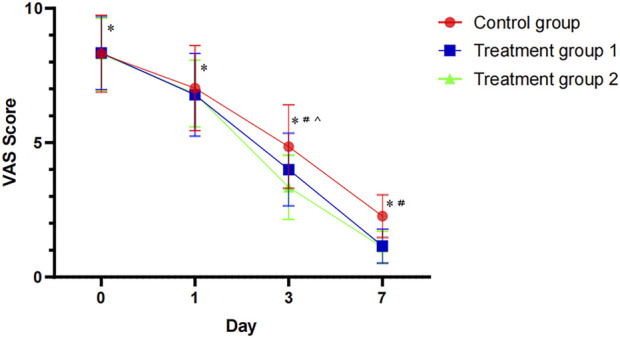
Comparison of the mean changes of patients' VAS score in the three groups. Statistical significant difference between before and after treatment in each group *P < 0.05; Statistical significant difference between treatment group 2 and control group ^#^P < 0.05; Statistical significant difference between treatment group 2 and treatment group 1 ^P < 0.05.

These results demonstrate that the combination of Jinhuang Ointment with conventional treatment was superior to the control group in both the magnitude and speed of pain relief. Furthermore, it provided a more pronounced and rapid analgesic advantage over the T1 group in the early phase of treatment (Figure 1).

### Time to onset of pain improvement in the target joint

**FIGURE 2:**
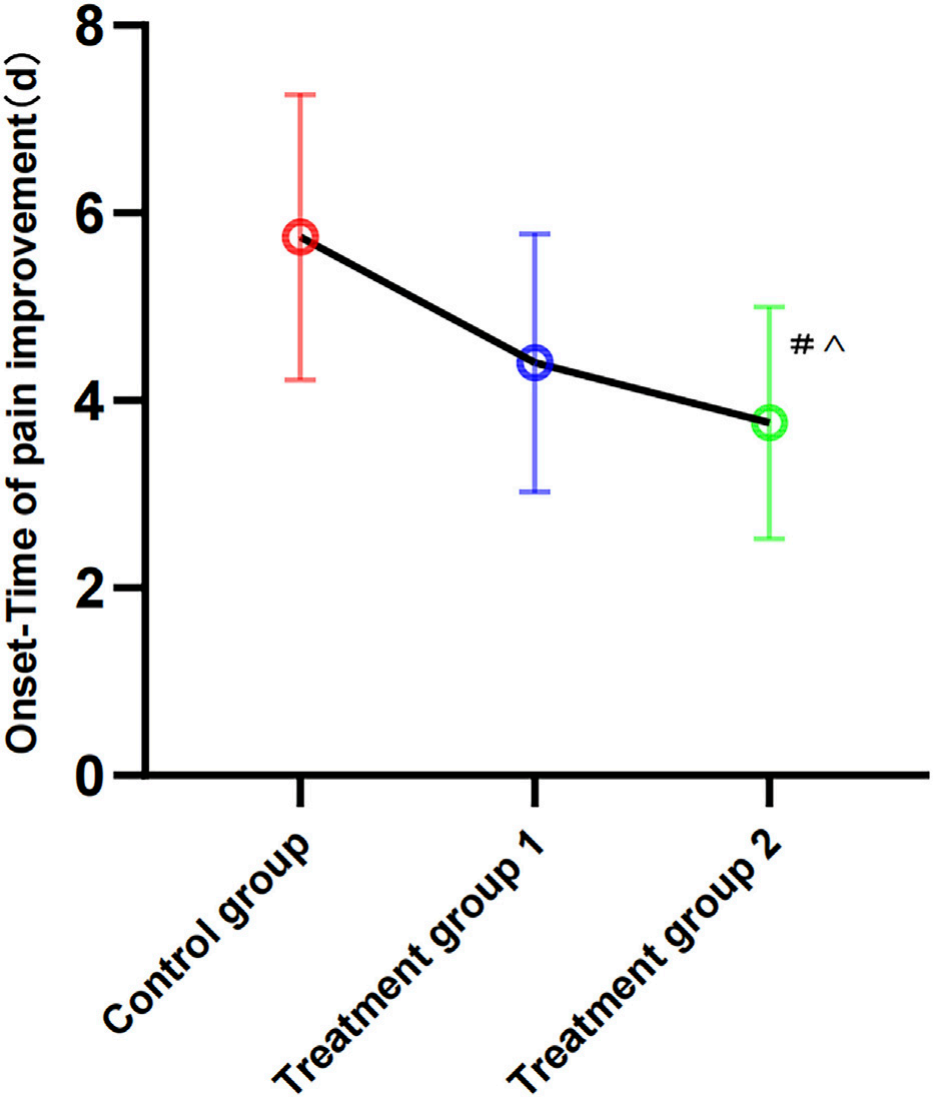
Comparison of onset-time of pain improvement of the target joint in three groups. Statistical significant difference between treatment group 2 and control group ^#^P < 0.05; Statistical significant difference between treatment group 2 and treatment group 1 ^P < 0.05. Changes in Joint Tenderness, Swelling, and Mobility Scores.

Using a VAS score <3 as the criterion for pain improvement, significant differences were observed in the time to onset among the three patient groups. Specifically, the T2 group demonstrated the shortest time to onset (3.76 ± 1.23 days, 3.335–4.195), which was significantly shorter than both the T1 group (4.40 ± 1.38 days, 3.927–4.873, P < 0.05) and the control group (5.74 ± 1.52 days, 5.220–6.265, P < 0.001). (Figure 2). These results indicate that Jinhuang Ointment can significantly accelerate the relief of pain symptoms in patients.

Before treatment, no statistically significant differences were observed in joint tenderness, swelling, or mobility scores among the three patient groups (P > 0.05). After 7 days of treatment, all three groups showed significant reductions in these scores compared to baseline (P < 0.01), with the T2 group demonstrating the most pronounced improvement (Table 3). Specifically, the T2 group achieved significantly lower joint tenderness (0.91 ± 0.379), swelling (0.53 ± 0.563), and mobility scores (0.56 ± 0.504) than the control group (1.40 ± 0.497, 0.94 ± 0.416, and 0.91 ± 0.284, respectively), with all differences being statistically significant (P < 0.01). Furthermore, compared to the T1 group, the T2 group exhibited superior improvement across all three dimensions. Notably, while no significant difference was observed between the T1 (0.74 ± 0.505) and control groups (0.74 ± 0.505) in joint swelling score, the T2 group (0.53 ± 0.563) showed a clear advantage (P < 0.01), further confirming the definite efficacy of Jinhuang Ointment in alleviating local joint symptoms and promoting functional recovery.

**TABLE 3:** The comparison of the scores for joint swelling and mobility function before and after treatment among the three groups ( $\bar{x}\pm S$ ) 7 days after treatment.

Items for evaluation	Treatment group 1 (n = 35)			Treatment group 2 (n = 34)			Control group (n = 35)		
	Before treatment	After treatment	95%CI	Before treatment	After treatment	95%CI	Before treatment	After treatment	95%CI
The score for joint tenderness	2.20 ± 0.759	0.89 ± 0.471*^#^	0.724–1.048	2.12 ± 0.64	0.91 ± 0.379*^#^	0.780–1.044	2.00 ± 0.642	1.40 ± 0.497*	1.229–1.571
The score for joint swelling	2.00 ± 0.642	0.74 ± 0.505*	0.569–0.917	2.03 ± 0.627	0.53 ± 0.563*^#^	0.333–0.726	1.97 ± 0.618	0.94 ± 0.416*	0.800–1.086
The score for joint mobility function	2.43 ± 0.558	0.60 ± 0.497*^#^	0.429–0.771	2.38 ± 0.551	0.56 ± 0.505*^#^	0.383–0.735	2.40 ± 0.604	0.91 ± 0.284*	0.817–1.012

Continuous data are presented as mean ± standard deviation (mean ± SD). *P < 0.05 indicates statistically significant differences within groups before and after treatment; #P < 0.05 indicates statistically significant differences between treatment group (T1 group or T2 group) and the control group.

### Changes in laboratory parameters

No statistically significant differences were observed in serum uric acid (UA), C-reactive protein (CRP), or erythrocyte sedimentation rate (ESR) levels among the three groups before treatment (P > 0.05). After 7 days of treatment, all three groups demonstrated significant reductions in these parameters compared to baseline (P < 0.05), with intergroup differences emerging. For ESR, the T2 group (14.794 ± 4.0360 mm/h) showed significantly lower values than the control group (24.26 ± 6.270 mm/h) (P < 0.05). Similarly for CRP, the T2 group (6.179 ± 2.8271 mg/L) exhibited significantly lower levels than the control group (10.103 ± 4.5622 mg/L) (P < 0.05). Although no statistically significant difference was found in UA levels, the T2 group (422.21 ± 69.153 μmol/L) displayed lower values than both the control group (440.43 ± 53.473 μmol/L) and the T1 group (434.71 ± 55.825 μmol/L) (Table 4). These results indicate that Jinhuang Ointment not only significantly lowers inflammatory markers (ESR and CRP) but also is associated with numerically reduced uric acid levels in the T2 group, despite the lack of statistical significance for the latter.

**TABLE 4:** Comparison of experimental indexes between the three groups before and after treatment ( $\bar{x}\pm S$ ) 7 days after treatment.

Item	Treatment group 1 (n = 35)			Treatment group 2 (n = 34)			Control group (n = 35)		
	Before treatment	After treatment	95%CI	Before treatment	After treatment	95%CI	Before treatment	After treatment	95%CI
ESR (mm/h)	42.14 ± 12.80	14.80 ± 3.571*^#^	13.573–16.027	42.26 ± 12.413	14.794 ± 4.036*^#^	13.386–16.202	44.14 ± 18.649	24.26 ± 6.270*	22.103–26.411
CRP (mg/L)	39.180 ± 19.5362	5.980 ± 2.4915*^#^	5.124–6.836	38.424 ± 22.2478	6.179 ± 2.8271*^#^	5.193–7.166	37.829 ± 20.5365	10.103 ± 4.5622*	8.536–11.670
UA (μmol/L)	470.86 ± 68.337	434.71 ± 55.825*	416.484–454.945	478.59 ± 70.939	422.21 ± 69.153*	398.077–446.334	474.57 ± 75.993	440.43 ± 53.473*	422.060–458.797

Continuous data are presented as mean ± standard deviation (mean ± SD). *P < 0.05 indicates statistically significant differences within groups before and after treatment; ^#^P < 0.05 indicates statistically significant differences between treatment group (T1 group or T2 group) and the control group.

### Safety evaluation

No severe adverse event was observed in the three groups. The incidence of adverse reactions was low across all three groups, with all cases being mild in severity. Specifically, the rates were 5.7% (2/35) in the control group, 11.4% (4/35) in the T1 group, and 5.9% (2/34) in the T2 group. All reported cases resolved shortly after simple symptomatic management without treatment discontinuation. No significant differences were observed in intergroup comparisons (P > 0.05) (Table 5). These results confirm that the addition of topical Jinhuang Ointment to conventional treatment maintains a favorable safety profile without introducing significant medication-related risks.

**TABLE 5:** Adverse reactions.

Item	Control group	Treatment group1	Treatment group2
Total adverse effects	2/35	4/35	2/34
Gastric or abdominal pain	2 (5.7%)	2 (5.7%)	1 (2.9%)
Edema	0	1 (2.9%)	0
Skin itch	0	1 (2.9%)	1 (2.9%)

Categorical data are presented as number (percentage) (n (%)).

### Post-hoc power analysis

To verify whether the actual sample size is sufficient to detect meaningful clinical effects, the “time to onset of pain improvement” (primary outcome indicator, defined as VAS score <3) was selected as the key index for power calculation. The analysis parameters were set based on the actual study results: the mean time to pain improvement was 3.76 ± 1.23 days in the T2 group (n = 34) and 5.74 ± 1.52 days in the control group (n = 35), with a mean difference of 1.98 days and pooled standard deviation of 1.38. The significance level (α) was set at 0.001 (two-tailed) according to the statistical results of intergroup comparison (P < 0.001).

The *post hoc* power analysis yielded a power value (1-β) of 0.99, which is far higher than the conventional threshold of 0.80. This indicates that the current sample size is sufficiently large to detect the true effect of Jinhuang Ointment on accelerating pain relief. Even in the retrospective design, the risk of type II error (false negative result) is extremely low, and the study conclusions are statistically reliable.

## Discussion

The prevalence of gouty arthritis has been steadily increasing worldwide, exhibiting a male predominance and a trend toward earlier onset (Singh and Gaffo, 2020; Zhang et al., 2022). This condition is closely associated with systemic comorbidities, including cardiovascular diseases, glucose metabolism disorders, and chronic kidney disease (Choi et al., 2022; Kim et al., 2015). Conventional management of acute gout relies on nonsteroidal anti-inflammatory drugs (NSAIDs) and colchicine (Wilson and Saseen, 2016; Yao et al., 2024). While effective for symptom control, their utility is often limited by adverse effects, significant interindividual variability in response, and contraindications in patients with underlying conditions (Ragab et al., 2017; Yao et al., 2024).

Guided by the principles of holism and syndrome differentiation, Traditional Chinese Medicine (TCM) offers distinct advantages in gout management, including consistent therapeutic effects, a favorable safety profile, and cost-effectiveness (Wang et al., 2024). This study evaluated the combination of topical Jinhuang Ointment with conventional Western treatment (T2 group) for acute gouty arthritis. The results demonstrated that the T2 group achieved superior improvements in VAS pain scores, joint swelling, tenderness, mobility, and inflammatory markers compared to the control group receiving conventional treatment alone. Notably, the T2 group also showed a significant advantage over both the T1 group (topical diclofenac diethylamine emulsion plus conventional treatment) and the control group in the time to pain relief and joint swelling scores. These findings suggest that integrating Jinhuang Ointment with baseline Western medication can accelerate the resolution of pain and swelling, effectively shortening the disease course and reducing symptom burden.

From the perspective of TCM theory, acute gouty arthritis is categorized as a “damp-heat obstruction pattern.” (Medicine SAoTC, 1994) Its pathogenesis involves deficiency of healthy qi and imbalance of yin and yang, leading to the accumulation of pathological products such as dampness, heat, phlegm, and stasis. These factors stagnate in the meridians and collaterals, and when combined with dietary irregularities, fatigue, or external pathogens, result in the blockage of qi and blood circulation, manifesting as gout. The core pathogenesis is thus damp-heat accumulation and blood stasis obstruction (Chi et al., 2020). Topical application of Chinese herbal medicine represents a safe, simple, and well-accepted treatment modality (Sun et al., 2023). This route bypasses gastrointestinal degradation and the hepatic first-pass effect, eliminates direct gastrointestinal irritation, and ensures drug absorption independent of digestive function (Huang et al., 2019). The primary therapeutic principle for topical gout treatment is “clearing heat and eliminating dampness.” Classic formulations such as Qingpeng Ointment (Shang et al., 2024), Qingluo Powder (Shu et al., 2022), and Qingbi Granules (Ren et al., 2020) are widely used for gout patients with damp-heat syndrome and have demonstrated satisfactory clinical outcomes.

Jinhuang Ointment, a common topical preparation in TCM surgery, functions to clear heat and detoxify, promote blood circulation to reduce swelling, and disperse nodules to relieve pain (Ye et al., 2022). This closely aligns with the pathogenesis of acute gouty arthritis. In this formula, *Trichosanthis Radix* (Tianhuafen) clears heat, promotes fluid production, detoxifies, and resolves abscesses, while raw *Arisaema Cum Bile* (Nanxing) dries dampness, transforms phlegm, dispels wind, and relieves convulsions. Their combination purges fire, eliminates dampness, expels wind, and activates the collaterals. *Phellodendri Chinensis Cortex* (Huangbo), with its bitter and cold properties, is particularly effective at clearing damp-heat from the lower energizer, making it a key herb for treating damp-heat pouring downward. It is combined with *Rhei Radix et Rhizoma* (Dahuang), *Angelicae Dahuricae Radix* (Baizhi), and *Curcumae Longae Rhizoma* (Jianghuang) to drain fire, remove toxins, clear heat, dry dampness, promote blood circulation and qi movement, and dispel stasis to relieve pain, together serving as minister herbs. Assisted by *Atractylodis Rhizoma* (Cangzhu), *Magnoliae Officinalis Cortex* (Houpo), and *Citri Reticulatae Pericarpium* (Chenpi)—which are bitter, warm, and aromatic to dry dampness—these herbs regulate qi, dry dampness, and strengthen the spleen, thereby cutting off the source of dampness and achieving the effect of “removing dampness to isolate heat.” *Glycyrrhizae Radix et Rhizoma* (Gancao) serves as the guiding herb, harmonizing the other components and assisting *Houpo* and *Chenpi* in boosting qi and fortifying the spleen. The combination of all herbs collectively achieves the effects of clearing heat, cooling the blood, resolving stasis, and relieving pain.

From a modern pharmacological perspective, the therapeutic actions of Jinhuang Ointment are likely mediated through multiple synergistic pathways, including anti-inflammation, immunomodulation, microcirculation improvement, and tissue repair promotion. The mechanisms may involve several aspects. First, regarding anti-inflammatory and immunomodulatory effects, curcumin from *Curcumae Longae Rhizoma* (Jianghuang) inhibits the NF-κB signaling pathway, reducing the release of inflammatory mediators such as IL-1β, IL-6, and TNF-α (Kocaadam and Sanlier, 2017). Pharmacological studies also indicate both Rhei Radix et Rhizoma (Dahuang) (Xu et al., 2024) and Phellodendri Chinensis Cortex (Huangbo) (Xian et al., 2011) exhibit significant anti-inflammatory activity. Furthermore, emerging evidence hypothesizes that the formula may modulate local immune responses by suppressing urate crystal-induced activation of the NLRP3 inflammasome, thereby controlling the production of key cytokines like IL-1β and mitigating acute inflammatory reactions (Li et al., 2023) These mechanisms may partially explain the improvement in inflammatory markers observed in this study. Second, concerning the improvement of local circulation and metabolism, components in Curcumae Longae Rhizoma (Jianghuang) and Atractylodis Rhizoma (Cangzhu) promote local blood circulation, accelerating the absorption and metabolism of inflammatory substances. Beyond its anti-inflammatory properties, Rhei Radix et Rhizoma (Dahuang) (Xu et al., 2024) promotes platelet aggregation and reduces antithrombin activity, contributing to local inflammation control and microcirculation improvement. This may be a key reason for the rapid resolution of swelling in the Jinhuang Ointment group. Third, the formula exhibits antioxidant and tissue repair effects. Phellodendri Chinensis Cortex (Huangbo) (Xian et al., 2011) and Atractylodis Rhizoma (Cangzhu) (Zhang et al., 2021) possess notable antioxidant activity, mitigating oxidative stress-induced tissue damage. Angelicae Dahuricae Radix (Baizhi) (Kang et al., 2008) exhibits strong astringent and tissue-repairing properties, enhancing local anti-infective capacity, promoting inflammation absorption and tissue regeneration. These effects may collectively contribute to the recovery of joint function. Collectively, the topical application of Jinhuang Ointment likely achieves rapid and significant clinical effects through multiple mechanisms, including antibacterial, anti-inflammatory, analgesic, and anticoagulant actions. Its anti-inflammatory effect alleviates local joint swelling, its analgesic effect helps relieve pain, and its anticoagulant property may assist in reducing edema by improving local microcirculation. This provides a plausible explanation for the finding that the time to pain improvement in the T2 group was significantly shorter than that in the T1 and control groups (P < 0.05), suggesting that Jinhuang Ointment may be superior to standard Western topical agents in achieving rapid early-stage pain relief and swelling reduction. It should be noted that these mechanisms are largely derived from studies on individual herbs or their components. Directly extrapolating these findings to the effects of the complete formula in humans remains insufficient. Future dedicated (preclinical) mechanistic studies on the complete Jinhuang Ointment formulation are necessary for confirmation.

Moreover, the findings of this study demonstrate that both the T1 and T2 groups exhibited significantly lower erythrocyte sedimentation rate (ESR) and C-reactive protein (CRP) levels compared to the control group, confirming that topical applications provide a distinct synergistic anti-inflammatory effect beyond the foundational impact of oral celecoxib. These changes in laboratory parameters offer critical support for the rationale of combination therapy: although the T1 and T2 groups showed markedly greater improvement in inflammatory markers than the control group, no significant differences in serum uric acid levels were observed among the three groups. This indicates that topical agents—including both Jinhuang Ointment and diclofenac diethylamine emulsion—alleviate symptoms primarily through local anti-inflammatory mechanisms rather than systemic regulation of uric acid. Further intergroup comparisons revealed that Jinhuang Ointment was not inferior to Western topical agents in controlling systemic inflammatory markers, while it demonstrated superior performance in the speed of pain relief and early swelling reduction, highlighting its advantage in comprehensive therapeutic efficacy. This observation can be explained by a synergistic mechanism of combined treatment: oral celecoxib provides systemic control of inflammatory activity, while topical agents achieve “targeted enhancement” through high local concentration at the lesion site, leading to more rapid and effective relief of acute symptoms such as joint redness, swelling, heat, and pain. Notably, the superior efficacy of Jinhuang Ointment in rapidly reducing swelling compared to diclofenac diethylamine emulsion may stem from the synergistic effects of its multi-component, multi-target mechanism—a characteristic feature of Chinese herbal medicine that emphasizes holistic regulation.

Regarding the safety profile, this retrospective analysis confirmed that Jinhuang Ointment was generally well-tolerated, with a low overall incidence of adverse events. Reported adverse reactions were predominantly mild dermal responses, such as localized pruritus or rash, which were generally manageable by reducing application time or adjusting drug concentration without requiring treatment discontinuation. The favorable safety profile is closely associated with its formulation technology. The rational selection of traditional bases such as vaseline, sesame oil, or lanolin helps minimize skin irritation, while the inclusion of modern permeation enhancers (e.g., azone) not only improves drug penetration but also further enhances local cutaneous tolerance (Williams and Barry, 2004). Compared with oral NSAIDs or colchicine, topical Jinhuang Ointment presents lower systemic risks owing to its localized action, rendering it particularly suitable for gout patients with comorbid gastrointestinal conditions or mild to moderate hepatic or renal impairment.

In summary, topical Jinhuang Ointment for acute gouty arthritis demonstrates multiple clinical advantages, including confirmed efficacy, rapid onset, favorable safety, and convenient application. Its mechanism of action appears to primarily involve local anti-inflammatory and anti-edema effects rather than systemic uric acid reduction. This topical route of administration avoids first-pass hepatic metabolism and gastrointestinal irritation associated with oral medications. It did not increase the risk of systemic adverse reactions in our study, demonstrates good patient compliance, and offers cost-effective operational simplicity. These attributes indicate a broad potential for its clinical adoption.

This study has several limitations. First, as a non-multicenter study, retrospective, non-randomized controlled study, its design carries inherent risks of selection bias and measurement bias. Although the baseline characteristics of the three patient groups were largely comparable, treatment allocation was based on clinical decision-making and a non-randomized process, leaving the possibility of unmeasured confounding factors. Furthermore, due to the distinct visual differences between Jinhuang Ointment and the control emulsion, blinding of both patients and outcome assessors was not feasible, which may have introduced potential bias in the assessment of subjective endpoints such as VAS pain scores, joint swelling, and tenderness. Second, the relatively modest sample size limits the statistical power of this study, which may have hindered the detection of subtle yet genuine differences between groups (such as in serum uric acid levels) and restricted more in-depth subgroup analyses. Consequently, the results, particularly any extrapolations, should be interpreted with appropriate caution. Additionally, the treatment and observation period was set at 7 days, focusing on short-term relief of acute-phase symptoms, which aligns with current clinical management principles for acute gout. However, this short-term design does not allow for the evaluation of the intervention’s impact on medium-to-long-term recurrence rates or long-term medication safety. It should be noted that this study focused on symptomatic management during the acute phase. Consistent with relevant guidelines, urate-lowering therapy was neither initiated nor adjusted during this stage to avoid precipitating “crystallization pain.” Thus, the omission of urate-lowering efficacy and recurrence rates as study endpoints is consistent with standard therapeutic logic for this disease phase. Finally, there remains room for further optimization and standardization of Jinhuang Ointment’s preparation process and dosage to enhance the stability and reproducibility of its therapeutic effects. Its precise pharmacological mechanisms, along with systematic pharmacokinetic and pharmacodynamic profiles, warrant further elucidation to provide a more solid scientific foundation for its modern clinical application.

Based on these limitations, future research should prioritize large-scale, multicenter, prospective, randomized, double-blind, controlled trials to better control for biases and validate the present findings. Concurrently, efforts should be made to establish standardized preparation protocols and quality control systems, extend follow-up duration to assess impacts on recurrence, and further explore the ointment’s molecular mechanisms. A systematic evaluation of its synergistic effects with other therapies and its value in the comprehensive management of gout is also recommended.

## Conclusion

This study demonstrates that the combination of topical Jinhuang Ointment with Western medicine produces synergistic effects in the treatment of acute gouty arthritis, significantly enhancing therapeutic efficacy and accelerating symptom relief, with a favorable safety profile. Future large-scale prospective randomized controlled trials are warranted to solidify its role in clinical practice.

## Data Availability

The original contributions presented in the study are included in the article/supplementary material, further inquiries can be directed to the corresponding authors.
